# Acupuncture for the treatment of ankle sprain: A protocol for a systematic review and meta-analysis: study protocol

**DOI:** 10.1097/MD.0000000000017905

**Published:** 2019-11-15

**Authors:** Fasen Huang, Kai Sun, Xuyue Pan, Kunming Xie, Junde Wu, Jingwei Tao, Yufeng Ma, Yinze Qi, Zhanhua Ma, Xinyu Li, Huan Liang, Shulong Wang, Zhen Lei, Zhaojun Chen

**Affiliations:** a The Third Affiliated Hospital of Beijing University of Chinese Medicine; b Wangjing Hospital, China Academy of Chinese Medical Sciences, Beijing, China.

**Keywords:** acupuncture, ankle sprain, protocol, systematic review

## Abstract

**Background:**

Ankle sprain is one of the most common musculoskeletal injuries in our daily life, which may lead to chronic ankle instability, reducing the quality of patients’ life and imposing a heavy burden on social medical security system. There are many kinds of methods treating ankle sprain, which can be divided into the conservative treatments and surgical intervention. Acupuncture is one of the conservative treatments for ankle sprain, especially in China. Therefore, we perform a systematic review and meta-analysis to evaluate the evidence for acupuncture's effectiveness, safety and cost benefits for the treatment.

**Methods:**

For the acquisition of required data of eligible randomized controlled trials (RCTs), literature search will be undertaken from the following database: PubMed, Embase, Web of Science, The Cochrane Library, Cochrane Central Register of Controlled Trials (CENTRAL), and ClinicalTrials.gov, Chinese National Knowledge Infrastructure (CNKI), VIP Database, and Wanfang database. Quality assessment of the included studies will be independently performed according to the Cochrane Risk of Bias Tool by 2 investigators and the level of evidence for results will be assessed using the Grading of Recommendations Assessment, Development, and Evaluation (GRADE) method. Statistical analysis will be conducted with Revman 5.3.

**Results:**

From the study we will assess the effectiveness, safety and cost benefit of acupuncture on pain relief and functional improvement in patients with ankle sprain.

**Conclusion:**

The conclusion of this study will provide evidence to ensure the effectiveness, safety and cost benefits of acupuncture on ankle sprain, which can further guide the selection of appropriate interventions.

**PROSPERO registration number:**

CRD42018116829.

## 1 Introduction

Ankle sprain is one of the most common musculoskeletal injuries in our daily life, with a rate of occurrence estimated at 10,000 ankle sprains per day in the USA, and it is estimated that almost 2 million people get this injury per year and this injury accounts for 20% of all sports injuries, especially in basketball sport.^[1–4]^ Those who participate in sports get a higher rate of ankle sprains than the general population and adolescents occur more frequently than adults, especially in high school.^[5–7]^ Ankle injuries are usually caused by an inversion and adduction of the foot in plantar flexion (supination), which mainly does damage to the lateral ankle, making up 85% of all occurrences.^[8–10]^ The main symptoms of ankle sprain conclude the patients’ complaint of pain on the outside of their ankle and various degrees of swelling and bleeding or bruising under the skin.^[8]^ According to the ligamentous damage and the associated degree of swelling and loss of range of motion (ROM), ankle sprains can be categorized into 3 grades from grade I to grade III: Grade I is the least severe one with mild stretching or partial tear of the anterior talofibular and/or calcaneofibular ligaments accompanied by mild tenderness and swelling but with slight or no functional loss; Grade II is incomplete tear of ligaments with moderate pain, swelling, and functional loss; And grade III is the most severe one characterized by complete tear of ligaments that results in severe swelling, pain, and loss of function and motion.^[11]^ If not treated promptly and effectively, it may leave a series of problems including persistent pain, chronic ankle instability, residual sensations of giving way, decrements in strength and the risk of posttraumatic ankle joint osteoarthritis and so on,^[12–14]^ which may result in high costs to society because of the increased health care resource use and work absence.^[15]^

The main object of ankle injury treatment is to control the acute inflammation process, relieve pain, regain full ankle range of motion (ROM), return to pre-injury level, increase muscle strength and power and prevent recurrence of injury.^[15,16]^ The 3 main treatments for ankle sprains are surgical treatment, conservative treatment involving immobilization with a plaster cast or splint, and functional conservative treatment with tape, a semi-rigid brace, or a lace-up brace plus coordination training.^[17–19]^ For the mild ankle sprain, the PRICE principal (protection, rest, ice, compression and elevation) is recommended while surgical treatment is needed for the severe injury.^[20]^ Also, there are many other treatments such as acetaminophen and nonsteroidal anti-inflammatory drugs (NSAIDs), and therapeutic ultrasonography, short-wave diathermy and neuromuscular electrical stimulation are also used in ankle sprains.^[21–23]^

Apart from the conventional treatments for ankle sprains, some complementary and alternative therapies such as herb, tuina and acupuncture have been used to help alleviate pain, reduce swelling, and speed up the recovery, but the evidence is limited. As an important component of traditional Chinese medicine, acupuncture has been widely reported to be used in the treatment of ankle sprains, and it showed an optimistic efficacy and less cost compared to other treatment.^[24–26]^ Some clinical experience in emergency department and even animal studies indicated that ankle sprain responded rapidly to acupuncture.^[27–30]^ As the efficacy of acupuncture for treating ankle sprains is optimistic but the evidence is limited, a critical examination of the evidence for the use of acupuncture for ankle sprains is warranted. Thus we decide to do this study to evaluate the evidence for acupuncture's effectiveness and safety for the treatment of ankle sprain.

## 2 Methods

### 2.1 Study registration

The protocol for this review has been registered in the International Prospective Register of Systematic Reviews (PROSPERO)(registration number: CRD42018116829) on December5, 2018. Available online: https://www.crd.york.ac.uk/PROSPERO/#myprospero. This protocol is reported in accordance with the Preferred Reporting Items for Systematic Review and Meta-Analysis Protocols (PRISMA-P) 2015 statement^[31]^ and the Cochrane Handbook for Systematic Reviews of Interventions.^[32]^

### 2.2 Inclusion criteria for study selection

#### 2.2.1 Type of studies

Only RCTs are included in our studies. Other designs, such as in vivo, in vitro, case reports, retrospective studies and non-RCTs will be excluded. There are no restrictions on languages.

#### 2.2.2 Type of participants

We will include studies on patients that are diagnosed as ankle sprain. The sex, age, race, severity, and duration of disease are not limited, and studies will be excluded if the participants get an ankle fracture or have other serious illnesses such as cancer, heart disease, liver disease, or kidney disease.

#### 2.2.3 Type of interventions

We will include the studies using acupuncture as the sole intervention in the experimental group, while we have no restrictions on intervention in the control group. Studies involving acupuncture combined with other therapies will be included if the other therapies are equally used in both experimental and control groups.

#### 2.2.4 Type of outcome measurements

*Primary outcomes*: Patient-reported global symptom improvement and visual analog scale (VAS) will be defined as the primary outcome at the end of the treatment, and pain intensity data would be included in the review if data for global symptoms are not provided.

*Secondary outcomes*:

(1) time to achieve pre-injury level of work or sports.
(2) subjective (e.g., giving way) and objective (e.g., inversion stress test, talar tilt, anterior drawer test, postural sway analysis).
(3) evaluations of ankle instability, dichotomous (e.g., yes or no) and continuous data regarding swelling, recurrence of ankle sprain, subsequent surgery, or long-term treatment.
(4) health-related quality of life (e.g., Short Form 36 (SF-36)).
(5) and adverse events related to acupuncture treatment.
(6) cost-effectiveness analysis, measured by any instrument.

### 2.3 Search strategy

An electronic search will be carried out in the following databases including the PubMed, Embase, Web of Science, The Cochrane Library, Cochrane Central Register of Controlled Trials (CENTRAL), ClinicalTrials.gov, Chinese National Knowledge Infrastructure (CNKI), VIP Database, and Wanfang data. No limits will be imposed on the dates, types, and statuses of the publications eligible for inclusion. The search strategy will include the following group terms: English (Ankle Injury OR Ankle Injuries OR Injury, Ankle OR Injuries, Ankle OR Ankle Sprain OR Ankle Sprains OR Sprain, Ankle OR Sprains, Ankle OR Syndesmotic Injury OR Syndesmotic Injuries OR Injury, Syndesmotic OR Injuries, Syndesmotic OR Ankle joint Injury OR Ankle joint Injuries OR Injury, Ankle joint OR Injuries, Ankle joint OR Ankle joint Sprains OR Ankle joint Sprain OR Sprain, Ankle joint OR Sprains, Ankle joint) AND (Acupuncture OR Acupuncture Treatments OR Treatment, Acupuncture OR Therapy, Acupuncture OR Pharmacoacupuncture Treatment OR Treatment, Pharmacoacupuncture OR Pharmacoacupuncture Therapy OR Therapy, Pharmacoacupuncture OR Acupotomy OR Acupotomies OR acupoint OR needling OR electroacupuncture OR electroacupuncture OR electric acupuncture OR hand acupuncture OR scalp acupuncture OR auricular acupuncture OR ear acupuncture) AND (randomized controlled trials OR controlled clinical trial OR randomized OR placebo OR clinical trials as topic OR randomly). Chinese (huai niu shang OR huai sun shang OR huai guan jie niu shang OR huai guan jie sunshang OR xia jing fei sun shang) AND (zhen jiu OR zhen ci OR er zhen OR dian zhen) AND (sui ji dui zhao shi yan OR lin chuang shi yan OR sui ji). Reference lists of the extracted studies will also be manually searched for further relevant articles.

### 2.4 Selection of studies

Two reviewers will independently read the title and abstract of the literature and screen the documents according to inclusion and exclusion criteria. When they are uncertain to determine whether to exclude, we will read the full text to identify the studies that need to be included. Any disagreements on study selection will be resolved through discussion with other researchers. We will report the study selection process according to the Preferred Reporting Items for Systematic Reviews and Meta-Analyses (PRISMA) guidelines.^[31]^

### 2.5 Data extraction

**Figure 1:**
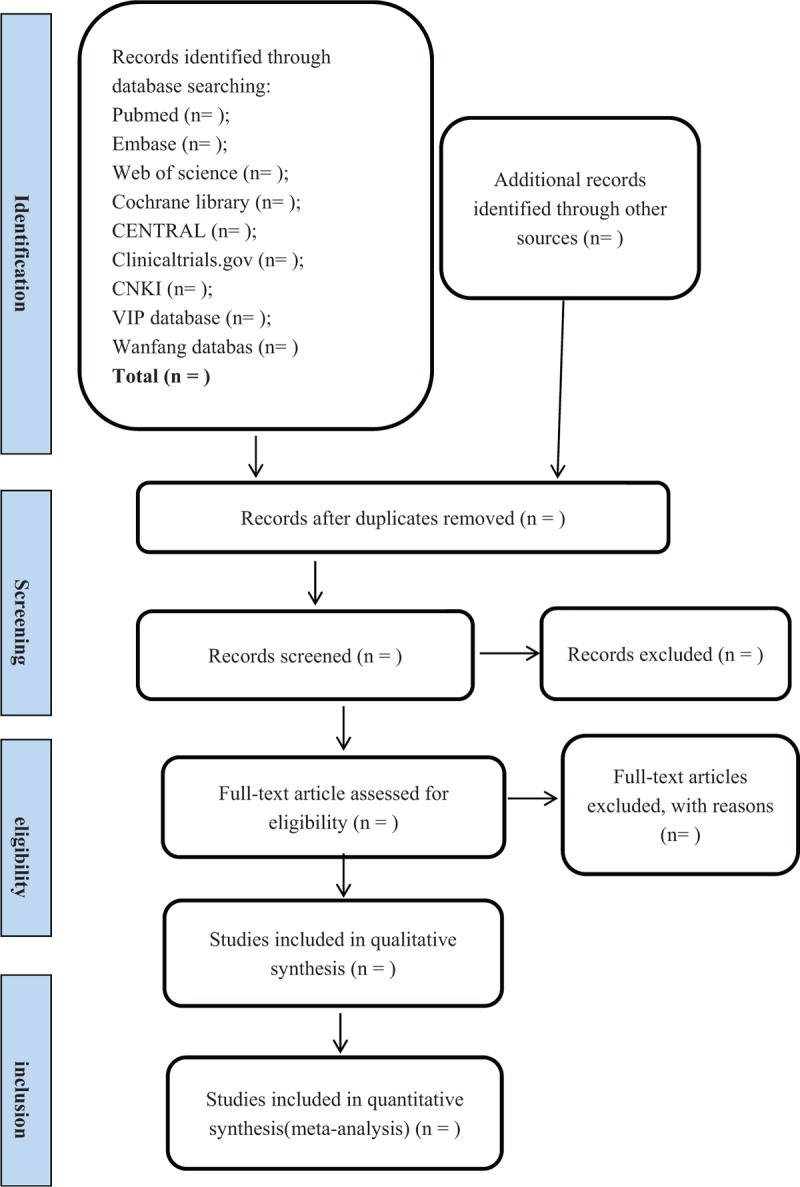
Flow diagram of literature search.

Two investigators will independently work for data extraction, and they will collect the following information:

(1) basic characteristics, including authors’ name, year of publication, country, study design, age and gender of patients, intervention, sample size, outcomes, adverse events, and follow-up. When relevant data is missing, we will contact the authors by email or in other ways to get the missing information.

(2) primary outcomes and secondary outcomes as the information showed before will also be included, including patient-reported global symptom improvement, pain intensity and so on. If there are some disagreements, the review authors will discuss or ask a third independent review author for conformation. The flow diagram of all study selection procedure is shown in Figure 1.

### 2.6 Assessment of risk of bias

Based on the Cochrane Handbook of Systematic Reviews of Interventions, the methodological quality of the included studies will be evaluated by 2 authors independently using the Cochrane risk of bias assessment tool. The domains to be assessed will include: random sequence generation, allocation concealment, blinding of participants and therapist, blinding of outcome assessment, incomplete outcome data, selective reporting, and other bias. The judgements on these items will be categorized as “low risk of bias”, “high risk of bias”, or “unclear risk of bias”. Discrepancies will be resolved by negotiation or by consulting other reviewers.

### 2.7 Measures of treatment effects

Dichotomous data and continuous variables are included in the outcomes of interest, and we will use the risk ratio (RR) to express dichotomous data use mean difference (MD) to assess differences in the continuous outcomes between the groups. Although different methods for the measurement of outcomes are used in different trials, we will choose standardized mean difference (SMD) if they have the same outcomes. The corresponding 95% confidence interval (CI) for each parameter will be calculated between the acupuncture treated group and the control group. We will choose descriptive review if quantitative synthesis is not appropriate.

### 2.8 Assessment of heterogeneity

*I*^2^ statistical test will be used to test the statistical heterogeneity of the included studies. If *I*^2^ ≥ 50%, which indicates the possibility of statistical heterogeneity, we will use a random-effects model for the calculation. Otherwise a fixed effects model will be used when the heterogeneity is not significant with its I^2^ < 50%.

### 2.9 Assessment of reporting bias

If more than 10 original studies are included in the meta-analysis, we will make funnel plots according to the data of the included studies to assess the bias such as publication bias. When reporting bias is implied by asymmetry of funnel plot, we will attempt to explain possible reasons. If the funnel plot is asymmetric, which indicates publication bias, we will discuss the sources and explain the possible reasons of bias.

### 2.10 Data synthesis

A forest plot for each parameter will be constructed to indicate the weight ratio of each incorporated study. All statistical analyses will be performed with the RevMan5.3 software, and the significance threshold will be *P* < .05 on 2-sides.

### 2.11 Sensitivity analysis

In order to evaluate the sensitivity and identify the robustness of the meta-analysis, we will perform sensitivity analyses by excluding:

(1) studies with high risks of bias and
(2) outliers that are numerically distant from the rest of the data.

### 2.12 Subgroup analysis

When heterogeneity is high, subgroup analyses will be performed for different comparators separately if the necessary. In addition, if the expected efficacy is not observed in all the subjects, the subgroup analysis could help us show whether the treatment is effective in certain subgroups. Also, subgroup analysis can help us show whether the therapeutic effect is better in particular subjects if it is found to be effective in all subjects.

### 2.13 Grading the quality of evidence

We will summarize the GRADE judgements according to the Grading of Recommendations Assessment, Development, and Evaluation (GRADE) method. The evidence quality evaluation of key outcome indicators can be can be classified into 4 levels: high (++++), moderate (+++), low (++), and very low (+) as recommended by the GRADE Working Group. Evidence quality is generally judged on the basis of risk of bias, inconsistency, indirectness, inaccuracy, and publication bias.

### 2.14 Ethics and dissemination

Because our study will not include animals or individuals, ethical approval will not be required. Once the results of the study are obtained, they will be published in conferences or peer-reviewed journals.

## 3 Discussion

Acupuncture therapy for ankle sprain has lots of advantages since it has higher acceptability and less pain, and it can reduce the use of other drugs, thereby decrease risks and costs. Although many clinical studies confirmed that acupuncture was effective for alleviating the symptoms of ankle sprain, there is no comprehensive systematic review giving enough evidence for such treatment. The protocol of this systematic review and meta-analysis study aims to assess the efficacy, safety and cost benefits of acupuncture in the treatment of ankle sprain. Meanwhile, we have tried our best to search and found that no relevant systematic review and meta-analysis concerning this topic has been reported in the last 5 years, and we will integrate the latest and most comprehensive clinical evidence in this field, hoping to offer a greater variety of treatment options for patients with ankle sprain and inspire more peer experts and doctors to carry out as many relevant studies as possible in the future. What is more, we hope our research can provide evidence and instruction for the spread and application of acupuncture treatment for ankle sprain in the future by analyzing and integrating the existing clinical studies. In addition, the systematic review and meta-analysis of GRADE evidence grading assessment are more conducive to clinical decision-making and guideline transformation. Meanwhile, this study has been registered on PROSPERO, which makes it more transparent and trustworthy. Of course, there are limitations in our study, because different types of acupuncture or different acupuncture points may lead to heterogeneity. We hope to make a more comprehensive, detailed and further study in the future.

## Author contributions

**Conceptualization:** Fasen Huang, Kai Sun.

**Data curation:** Xuyue Pan, Kunming Xie, Jingwei Tao.

**Methodology:** Zhaojun Chen.

**Resources:** Huan Liang, Shulong Wang, Zhen Lei.

**Software:** Junde Wu, Zhanhua Ma.

**Supervision:** Yufeng Ma, Yinze Qi, Xinyu Li.

**Writing – original draft:** Fasen Huang, Kai Sun.

**Writing – review & editing:** Fasen Huang, Kai Sun, Zhaojun Chen.
